# Evaluation of the association between periodontitis and risk of Parkinson’s disease: a nationwide retrospective cohort study

**DOI:** 10.1038/s41598-021-96147-4

**Published:** 2021-08-16

**Authors:** Eunkyung Jeong, Jun-Beom Park, Yong-Gyu Park

**Affiliations:** 1 Department of Biomedicine & Health Science, Graduate School, The Catholic University of Korea, Seoul, 06591 Republic of Korea; 2 Department of Periodontics, College of Medicine, The Catholic University of Korea, 222, Banpo-daero, Seocho-gu, Seoul, 06591 Republic of Korea; 3 Department of Medical Lifescience, College of Medicine, The Catholic University of Korea, 222, Banpo-daero, Seocho-gu, Seoul, 06591 Republic of Korea

**Keywords:** Medical research, Epidemiology

## Abstract

The objective of this study was to examine the association between periodontitis and risk of incident Parkinson’s disease using large-scale cohort data on the entire population of South Korea. Health checkup data from 6,856,180 participants aged 40 and older were provided by the National Health Insurance Service of South Korea between January 1, 2009, and December 31, 2009, and the data were followed until December 31, 2017. The hazard ratio (HR) of Parkinson’s disease and 95% confidence interval (CI) were estimated using a Cox proportional hazards model adjusted for potential confounders. The incidence probability of Parkinson’s disease was positively correlated with the presence of periodontitis. The HR of Parkinson’s disease for the participants without the need of further dentist visits was 0.96 (95% CI 0.921–1.002); the HR of Parkinson’s disease increased to 1.142 (95% CI 1.094–1.193) for the individuals who needed further dentist visits. Compared to individuals without periodontitis and without metabolic syndrome, the HR of incident Parkinson’s disease gradually increased for individuals with periodontitis, with metabolic syndrome, and with both periodontitis and metabolic syndrome. People with periodontitis and metabolic syndrome had the highest HR of incident Parkinson’s disease, at 1.167 (95% CI 1.118–1.219). In conclusion, a weak association between periodontitis and Parkinson’s disease was suggested after adjusting for confounding factors from the population-based large-scale cohort of the entire South Korean population.

## Introduction

Parkinson’s disease is a progressive neurodegenerative disease associated with loss or insolvency of dopaminergic neurons in the brain^1^. Parkinson’s is the second most common neurodegenerative disease in the world, after Alzheimer’s disease^2^. Parkinson’s disease is characterized by rigidity, akinesia and tremor^3^, and it leads to memory loss, cognitive decline and dementia^4^. There has been growing awareness of the non-motor symptoms of Parkinson’s disease over the past decade^5^. Parkinson’s disease hinders the automatic movement of the hands, which can affect oral hygiene and care^3^. Some of the resulting conditions include higher prevalence of dental caries, periodontitis, sialorrhea, xerostomia, and impairment of taste^5^.

Systemic medical conditions can negatively affect oral health^6^. Dental disease and Parkinson’s disease have been linked to problems in oral health due to poor exercise and cognitive control among patients with Parkinson’s disease^1,7^. Lower frequencies of daily toothbrushing and educed salivary flow were seen in Parkinson’s patients^8,9^. Patients with Parkinson’s disease have significantly more cariogenic bacteria in their saliva^10^. Higher number of dental caries and higher tooth loss frequency is seen with patients with Parkinson’s disease than in the control group^1^. The number of Parkinson’s patients visiting dental clinics to be treated with complete dentures is increasing^11^. Previous report showed that the number of tooth loss was positively correlated with a higher risk of development of newly onset Parkinson's disease in a longitudinal study setting^12^. Increased susceptibility to periodontitis in Parkinson’s disease is reported in the individuals with Parkinson’s disease^11^. Statistically significant differences were observed between patients with Parkinson’s disease and a control group in probing depth, bleeding on probing, gingival index, and plaque index^13^. There is limited report evaluating the link between the Parkinson’s disease outbreak and periodontitis^14^. Therefore, the purpose of this study was to determine the association of periodontitis and the risk of incident Parkinson’s disease using large-scale cohort data for the entire South Korean population.

## Materials and methods

### Study population

This study used the entire population-based database provided by the National Health Insurance Service (NHIS) of South Korea. The NHIS is a single insurer supervised by the Ministry of Health and Welfare of the South Korean government^15^. This database has extensive health information, including sociodemographic information, use of medical treatments, and pharmacy-dispensing claims. Diseases were diagnosed based on 10th Revision of the Clinical Modification of International Classification of Diseases (ICD-10). From this database, 10,505,818 individuals had received health checkups from the NHIS once or more from January 1, 2009, to December 31, 2009. We excluded patients younger than 40 years old, resulting in 7,183,262 participants. We excluded the individuals whose data were missing, resulting in 6,856,180 participants. The participants with Parkinson’s disease were excluded, resulting in 6,843,261 patients. We excluded individuals who were diagnosed with Parkinson’s disease within 1 year after the enrollment. Parkinson’s disease was diagnosed based on based on ICD-10 code for PD (G20) and the registration code for PD (V124) in the program implemented by NHIS to enhance the health coverage for rare intractable diseases including Parkinson’s disease since the year of 2006^16^. It should be noted that all patients with rare intractable diseases like Parkinson’s disease had their diagnosis certified by a physician according to uniform diagnostic criteria used by the NHIS^17^. A total of 6,825,684 participants (3,427,327 males and 3,398,357 females) were included in the study and were tracked up to December 21, 2017, or up to their deaths. This study was performed according to the Declaration of Helsinki and was approved by the Institutional Review Board of Seoul St. Mary’s Hospital, Catholic University of Korea, South Korea (KC19ZESI0422). A waiver to the requirement of written informed consent was approved due to the information being anonymous and not involving human rights violations or ethical infringement.

### Assessment and definitions

Health examinations were performed to measure height, weight, waist circumference, and blood pressure. Body mass index was defined as the individual’s weight (kg) divided by the individual’s height squared (m^2^). Systolic blood pressure and diastolic blood pressure were measured after resting for at least 5 min in a seated position. Laboratory tests were done to evaluate fasting glucose, total cholesterol, gamma-glutamyl transferase, and triglycerides, high-density lipoprotein cholesterol and low-density lipoprotein cholesterol. Metabolic syndrome was defined following the modified criteria of the National Cholesterol Education Program Adult Treatment Panel III, while the Asian-specific waist circumference cutoff was applied for abdominal obesity^18^. In short, participants having with at least three of the following components were diagnosed as metabolic syndrome: (1) waist circumference 90 cm for men or 85 cm for women; (2) serum triglycerides 17.0 mmol/dL or treatment with lipid-lowering medication; (3) serum high-density lipoprotein cholesterol < 10.4 mmol/dL for men or < 13.0 mmol/dL for women or treatment with lipid-lowering medication; (4) systolic blood pressure 130 mm Hg, diastolic blood pressure 85 mm Hg, or treatment with anti-hypertensive medication; and (5) fasting plasma glucose of 55.5 mmol/dL or use of hypoglycemic agents.

More information on the participants’ lifestyles was obtained through standardized self-reporting questionnaires, such as on smoking status, alcohol consumption, exercise habits, and income. Smoking status was dichotomized as being a current smoker, and heavy drinkers were defined as consuming ≥ 30 g of alcohol per day^19^. Regular exercise was defined as strenuous or moderate exercise at least once per week, and low income level was defined as the lowest 20% of the population^20^. Patients were classified into the periodontitis group when ICD-10 code for periodontitis (K05.3) was identified at the index examination. The periodontitis group was divided into three disease severity stages (as mild, moderate and severe periodontitis), according to the severity of periodontitis, based on therapeutic procedures including scaling, subgingival curettage, and periodontal surgery^21^. Individuals without periodontitis were considered the non-periodontitis group.

### Statistical analysis

The SAS software package was used for the statistical analyses (version 9.3; SAS Institute, Cary, NC, USA). The baseline characteristics of the study participants based on the presence of periodontitis were given in terms of the mean ± standard deviation for continuous variables or the number (percentage) for categorical variables. Independent *t*-tests were used to compare the continuous variables, and chi‐square tests were used for the categorical variables. Kaplan–Meier curves were the schematics used to indicate the cumulative incidence probability of Parkinson’s disease, and log‐rank testing was performed to assess the differences in the effects of periodontitis on the development of Parkinson’s disease. The incidence rate of Parkinson’s disease was calculated by dividing the number of events by the number of person‐years. Cox proportional hazards analyses were performed to assess the association between periodontitis and incidence of Parkinson’s disease, and hazard ratios (HRs) and 95% confidence intervals (CIs) were calculated. Model 1 was adjusted for age and sex. Model 2 was adjusted for the values in Model 1 plus smoking status, drinking habits, exercise habits, income level, body mass index, diabetes mellitus, hypertension, and dyslipidemia. Model 3 was adjusted for the values in Model 2 plus stroke and depression. Additionally, 3-year and 5-year lagged analyses were performed on the Model 3. The *P* value of 0.05 was considered to be statistically significant.

## Results

### Baseline characteristics

The baseline characteristics of the study population according to the presence of periodontitis are shown in Table 1. At baseline, 903,063 individuals (13.2% of the total population) were diagnosed with periodontitis. The mean age was 55.47 ± 9.97 years in the periodontitis group and 54.21 ± 10.54 in the group without periodontitis. The proportion of men was higher in the periodontitis group than in the non-periodontitis group. Individuals with periodontitis exhibited higher mean body mass index, waist circumference, fasting glucose, gamma-glutamyl transferase, and triglycerides. The proportion of current smokers was higher in the periodontitis group than in the non-periodontitis group, and participants with periodontitis had higher prevalence of diabetes mellitus, hypertension, and dyslipidemia.

**Table 1:** Baseline characteristics of the study population according to the presence of Parkinson’s disease.

Characteristics	Without periodontitis	With periodontitis	*P*-value
n	5,922,621	903,063	
Age (years)	54.21 ± 10.54	55.47 ± 9.97	< 0.0001
**Sex**			< 0.0001
Male	2,941,386 (49.66)	485,941 (53.81)	
Female	2,981,235 (50.34)	417,122 (46.19)	
Body mass index (kg/m^2^)	23.96 ± 3.03	24.08 ± 2.96	< 0.0001
Waist circumference (cm)	81.14 ± 8.58	81.85 ± 8.47	< 0.0001
Systolic blood pressure (mm Hg)	124.23 ± 15.46	124.2 ± 15.16	0.0547
Diastolic blood pressure (mm Hg)	77.2 ± 10.17	77.09 ± 10.01	< 0.0001
Fasting glucose (mmol/dL)	99.71 ± 24.66	101.27 ± 26.33	< 0.0001
Total cholesterol (mmol/dL)	199.09 ± 36.97	199.04 ± 37.2	0.253
Gamma-glutamyl transferase (IU/L)	24 (16–40)	25 (17–42)	< 0.0001
Triglycerides (mmol/dL)	114 (80–168)	117 (82–171)	< 0.0001
Current smoker	1,236,162 (20.87)	192,985 (21.37)	< 0.0001
Heavy drinker	369,309 (6.24)	56,683 (6.28)	0.1318
Regular exercise	2,930,465 (49.48)	467,168 (51.73)	< 0.0001
Low income (lowest 20%)	1,313,890 (22.18)	188,015 (20.82)	< 0.0001
Diabetes mellitus	673,345 (11.37)	126,492 (14.01)	< 0.0001
Hypertension	1,981,598 (33.46)	323,187 (35.79)	< 0.0001
Dyslipidemia	1,350,485 (22.8)	229,034 (25.36)	< 0.0001
Stroke	131,482 (2.22)	25,376 (2.81)	< 0.0001
Depression	242,827 (4.1)	46,146 (5.11)	< 0.0001

The data were presented as the mean ± standard deviation for continuous variables or the number (percentage) for categorical variables.

### Incidence probability of Parkinson’s disease according to the presence and severity of periodontitis

**Figure 1:**
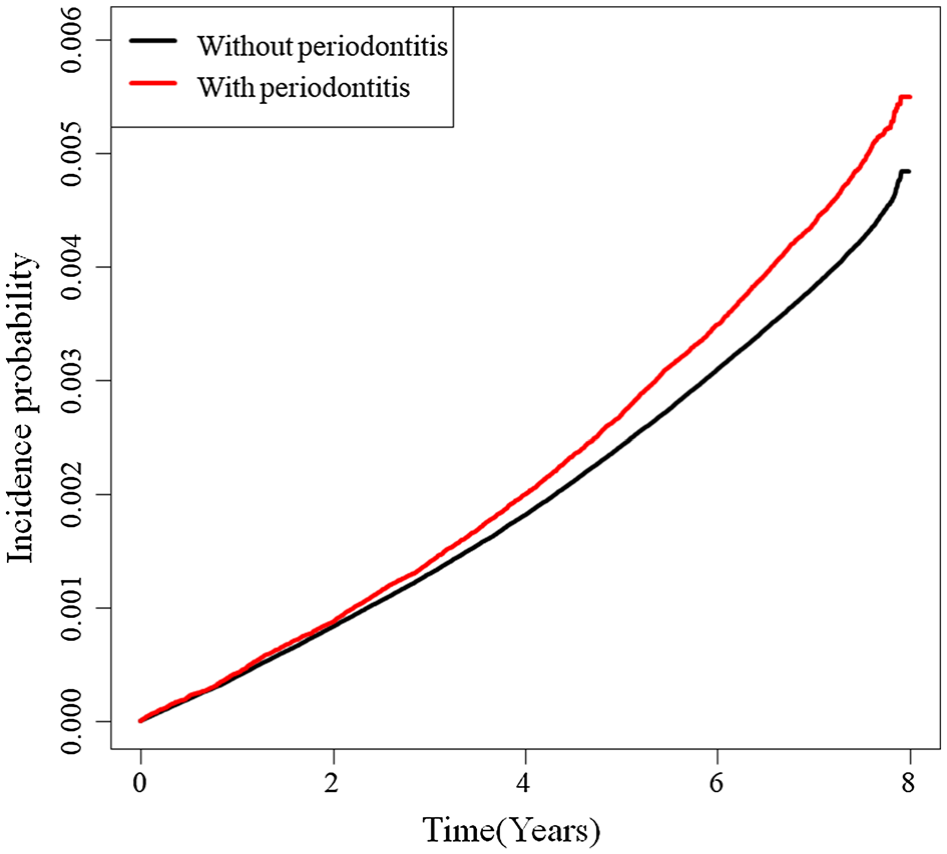
Kaplan–Meier curves of the incidence probability for Parkinson’s disease categorized by the presence of periodontitis. The incidence probability of Parkinson’s disease was higher with the presence of periodontitis (log rank *P* < 0.001).

Figure 1 demonstrates the Kaplan–Meier curves of the probability of incidence of Parkinson’s disease for up to 8 years according to the presence of periodontitis, as compared to the group without periodontitis. The incidence probability of Parkinson’s disease was positively correlated with the presence of periodontitis (log-rank *P* < 0.001).

### Longitudinal association between the presence and severity of periodontitis and incidence rate of Parkinson’s disease

Table 2 shows the longitudinal association between the severity of periodontitis and incidence of Parkinson’s disease. After adjusting for age and sex in Model 1, the incidence of Parkinson’s disease in the whole population was increased due to the participants with periodontitis. The association between periodontitis and Parkinson’s disease and the incidence of Parkinson’s disease persisted after further adjustment for smoking status, drinking habits, exercise habits, income level, body mass index, diabetes mellitus, hypertension, and dyslipidemia. In the subgroup analysis, the HR of Parkinson’s disease was 1.088 (95% CI 1.049–1.128) for participants with mild periodontitis after adjustment (Model 2). In Model 3 with additional adjustment for stroke and depression, the HR of Parkinson’s disease was 1.095 (1.063–1.128) for participants with mild periodontitis. However, the HRs of Parkinson’s disease given moderate and severe periodontitis were 1.069 (95% CI 0.997–1.148) and 0.999 (95% CI 0.829–1.204), respectively for Model 2. The HRs of Parkinson’s disease given moderate and severe periodontitis in Model 3 are 1.076 (95% CI 1.005–1.153) and 0.987 (95% CI 0.82–1.188), respectively. The effects of follow-up visits within 1 year after periodontitis treatment on the incidence of Parkinson’s disease were evaluated. The HR of Parkinson’s disease for the participants with follow-up visits was 1.078 (95% CI 1.042–1.115), which increased to 1.136 (95% CI 1.015–1.272) for the individuals without follow-up visits for Model 2. The HR of Parkinson’s disease for the participants the need of further visits was 1.078 (95% CI 1.04–1.119), which increased to 1.114 (95% CI 1.062–1.146) for the individuals who needed further visits for Model 3.

**Table 2:** Longitudinal association between the severity of periodontitis and incidence of Parkinson’s disease.

Variables	n	Events	Person-years	Incidence rate per 1000 person-years	Model 1	Model 2	Model 3	Model 3 (3-year lag)	Model 3 (5-year lag)
**Periodontitis**									
No	5,922,621	24,034	42,772,055.50	0.56191	1 (reference)	1 (reference)	1 (reference)	1 (reference)	(reference)
Yes	903,063	4,245	6,532,573.13	0.64982	1.099 (1.064, 1.136)	1.082 (1.047, 1.118)	1.090 (1.06,1.121)	1.103 (1.069,1.138)	1.109 (1.068,1.151)
**Type of periodontitis**									
None	5,922,621	24,034	42,772,055.50	0.56191	1 (reference)	1 (reference)	1 (reference)	1 (reference)	1 (reference)
Mild	660,675	3,331	4,771,056.49	0.69817	1.103 (1.063, 1.143)	1.088 (1.049, 1.128)	1.095 (1.063,1.128)	1.102 (1.066,1.139)	1.101 (1.059,1.263)
Moderate	212,612	803	1,545,323.43	0.51963	1.095 (1.021, 1.175)	1.069 (0.997, 1.148)	1.076 (1.005,1.153)	1.127 (1.045,1.215)	1.156 (1.059,1.263)
Severe	29,776	111	216,193.21	0.51343	1.026 (0.852, 1.237)	0.999 (0.829, 1.204)	0.987 (0.82,1.188)	0.999 (0.813,1.228)	1.088 (0.86,1.377)
**Visit one year after treatment of periodontitis**									
No periodontitis	5,922,621	24,034	42,772,055.50	0.56191	1 (reference)	1 (reference)	1 (reference)	1 (reference)	1 (reference)
No re-visit	844,743	3,938	6,109,817.15	0.64454	1.094 (1.058, 1.132)	1.078 (1.042, 1.115)	1.078 (1.04,1.119)	1.099 (1.055,1.144)	1.119 (1.067,1.174)
Re-visit	58,320	307	422,755.98	0.72619	1.165 (1.041, 1.304)	1.136 (1.015, 1.272)	1.114 (1.062,1.146)	1.108 (1.062,1.157)	1.097 (1.042,1.154)

Model 1 was adjusted for age and sex.

Model 2 was adjusted for the values in Model 1 plus smoking status, drinking habits, exercise habits, income level, body mass index, diabetes mellitus, hypertension, and dyslipidemia.

Model 3 was adjusted for the values in Model 2 plus stroke and depression.

### Subgroup analysis for risk of periodontitis

Table 3 shows the results for the subgroup analysis for periodontitis with adjustments for age, sex, smoking status, drinking habits, exercise habits, income level, body mass index, diabetes mellitus, hypertension, dyslipidemia, stroke and depression. The HRs for periodontal disease were 1.001 (95% CI 0.955–1.049) and 1.077 (95% CI 1.04–1.115) for individuals < 65 years old and ≥ 65 years old, respectively (*P* < 0.05). The HRs of men and women with periodontal disease were 1.065(95% CI 1.024–1.108) and 1.114(95% CI 1.07–1.158), respectively.

**Table 3:** Subgroup analysis for periodontitis.

Variables	Severity of periodontitis	
	Hazard ratios (95% confidence interval)	*P* for interaction
**Age (years)**		
< 65	1.001 (0.955, 1.049)	0.0945
≥ 65	1.077 (1.04, 1.115)	
**Sex**		
Male	1.065 (1.024, 1.108)	0.0677
Female	1.114 (1.07, 1.158)	
**Obesity**		
No	1.103 (1.064, 1.143)	0.1032
Yes	1.061 (1.014, 1.111)	
**Abdominal obesity**		
No	1.09 (1.053, 1.128)	0.8534
Yes	1.087 (1.035, 1.141)	
**Diabetes mellitus**		
No	1.091 (1.057, 1.126)	0.7721
Yes	1.084 (1.023, 1.149)	
**Hypertension**		
No	1.104 (1.057, 1.152)	0.275
Yes	1.074 (1.035, 1.114)	
**Dyslipidemia**		
No	1.096 (1.059, 1.134)	0.3422
Yes	1.078 (1.027, 1.132)	
**Smoking**		
No	1.088 (1.056, 1.121)	0.6522
Yes	1.106 (1.017, 1.204)	
**Drinking**		
None	1.09 (1.059, 1.121)	0.9025
Heavy drinker	1.101 (0.965, 1.256)	
**Stroke**		
No	1.094 (1.062, 1.126)	0.4564
Yes	1.036 (0.946, 1.135)	
**Depression**		
No	1.092 (1.06, 1.125)	0.863
Yes	1.076 (0.997, 1.16)	

The model was adjusted for age, sex, smoking status, drinking habits, exercise habits, income level, body mass index, diabetes mellitus, hypertension, dyslipidemia, stroke and depression.

The hazard ratios are adjusted for age, sex, smoking history, drinking history, exercise habits, income level, body mass index, diabetes mellitus, hypertension, dyslipidemia, stroke and depression.

### Risk of incident Parkinson’s disease according to the combination of periodontitis and metabolic syndrome

**Figure 2:**
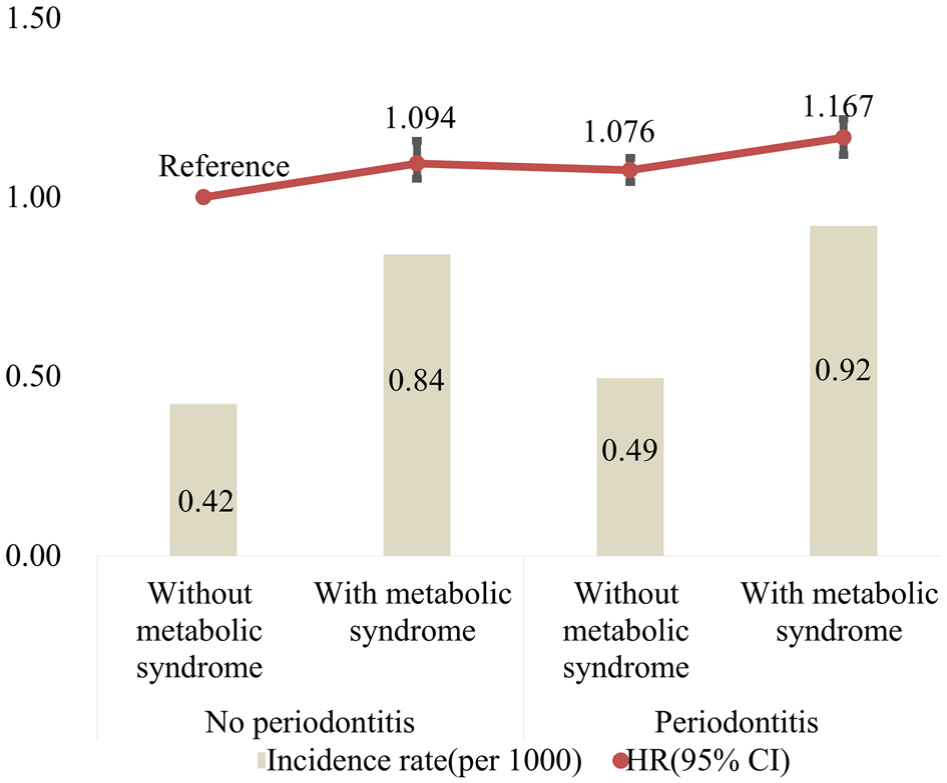
The combined effects of periodontitis and metabolic syndrome on incident Parkinson’s disease risk after adjusting for confounding factors. Periodontitis and metabolic syndrome had a significant interaction on the risk of Parkinson’s disease (*P* for interaction < 0.001).

Figure 2 shows the combined effects of periodontitis and metabolic syndrome on incident Parkinson’s disease risk after adjustments for confounding factors. Periodontitis and metabolic syndrome had a significant interaction on the risk of Parkinson’s disease (*P* for interaction < 0.001). As compared to individuals without periodontitis and without metabolic syndrome, gradual increases in the HR of incident Parkinson’s disease were observed for individuals with periodontitis, with metabolic syndrome, and with periodontitis and metabolic syndrome. People with periodontitis and metabolic syndrome had the highest HR of incident Parkinson’s disease, at 1.167 (95% CI 1.118–1.219).

## Discussion

The association between periodontitis and the development of Parkinson’s disease has not yet been fully investigated. The objective of this study was to assess the association of periodontitis with the risk of incident Parkinson’s disease using large-scale cohort data for the entire South Korean population.

Increasing evidence has suggested an independent association between periodontitis and various comorbidities, including diabetes mellitus, cardiovascular disease, respiratory disease, and rheumatoid arthritis^22,23^. The effects of Parkinson’s disease on periodontal diseases have also been reported^24^ and Parkinson’s disease is considered a periodontitis comorbidity with these diseases being linked by low-grade systemic inflammations^25,26^. Indeed, systemic inflammation has been suggested to be one of the causes of neurodegeneration^7^ and individuals with Parkinson’s diseases had higher prevalence for periodontitis and the periodontal clinical parameters deteriorated with increase in the severity of Parkinson’s disease^13^.

Bidirectional interrelationships between periodontal diseases and systemic diseases have been suggested in previous reports^27^. This study evaluated the longitudinal association between the severity of periodontitis and incidence of Parkinson’s disease and the association was clearly seen after adjustment of confounding factors. Similarly, a previous report showed that patients with periodontal inflammatory disease had a higher risk of developing Parkinson’s disease, with an adjusted HR of 1.43^14^. There are contradictory results. It has been shown that poor oral health was not related with the occurrence of Parkinson’s disease or with the number of natural teeth or oral mucosal lesions^28^. In another report, the candida-related oral mucosal lesions were associated with Parkinson’s disease in men, showing an HR of 1.56^28^. It can be emphasized that periodontal treatment reduces the inflammation in oral cavity and this may produce positive effects reducing the inflammation in the body^29^. The protective effect of dental follow-up among individuals with Parkinson’s disease may be suggested. Previous report showed that longer time since the last dentist visit was seen in Parkinson’s patients^8^. The current study showed that the incidence of Parkinson’s disease was higher among individuals who needed a follow-up dental visit, with an HR of 1.142. The protective effects of dental scaling were evaluated, and the results showed that the patients without periodontal inflammatory disease who underwent dental scaling over five consecutive years had a significantly lower incidence of Parkinson’s disease^30^. Moreover, the development of Parkinson’s disease was lower for the people receiving scaling with periodontitis when compared with the participants receiving scaling without periodontitis^30^. It can be suggested that the treatment and prevention of periodontal diseases may benefit systemic health.

The role of host factors in Parkinson's disease and periodontitis should also be considered. The prevalence of Parkinson’s disease is reported to increase steadily with age^31^. Similarly, the prevalence of periodontal disease is shown to increase with the advancing age^32^. Sex differences between females and males were seen in the prevalence of Parkinson’s disease, with male participants aged 50–59 having with a higher prevalence^31^. The cutoff value of age for moderate and severe periodontitis was 43 years in men and 49 years in women^33^. The ethnicity of the participants may affect the prevalence of Parkinson’s disease^34^. It was shown that age-standardized prevalence of Parkinson disease was higher in Whites than in Asians. It was shown that the severity of periodontitis may vary in different population including regional differences^35,36^. It should be noted that adjustments were made with various variables including age, sex, smoking status, drinking habits, exercise habits, income level, and body mass index in this study. Previous report showed that incidence of Parkinson’s disease was positively correlated with the number of metabolic syndrome components^37^. Risk of incident Parkinson’s disease according to the combination of periodontitis and metabolic syndrome showed that participants with periodontitis and metabolic syndrome had the highest HR of incident Parkinson’s disease, at 1.167.

Oral diseases are prevalent in people with Parkinson’s disease, but patients, physicians, and caregivers often neglect the patients’ oral-health needs ^5^. People with Parkinson’s disease pose a challenge to dentists because the degenerative disease causes problems in patients accessing dental care and maintaining adequate oral health^38^. It has been widely shown that the psychological components of Parkinson’s disease include cognitive deficiency and that there is a great need to encourage dental plaque control^1,9,11,13^. Increased cooperation from dentists and physicians is needed to ensure optimal screening, treatment, and prevention of periodontitis and Parkinson’s disease^22^.

The present study contains several limitations. The NHIS provides biennial health evaluations, but half of the target participants received the health evaluation voluntarily^15^. The participants may have healthier habits when compared with uncooperative participants^39^. Furthermore, misdiagnosis from underestimating or overestimating Parkinson’s disease may have been possible. There is another possibility that the symptoms may have started some time before the diagnosis of Parkinson’s disease^40^. In this study, annual incidence rates were used to minimize the issue of periodontitis having a time varying characteristics^41^. Periodontitis develops over time and it can be treated, but it may be very difficult to have it treated completely^32,42^. We tried to divide the periodontitis group based on the severity based on the therapeutic procedures including scaling, subgingival curettage, and periodontal surgery. There is only weak association between periodontitis and Parkinson’s disease because the HR dropped with more severe type of periodontitis, showing inverse U-shape association. The study, which used the NHIS database, was not designed prospectively^43^. However, this study uses the diagnosis of Parkinson’s disease from the NHIS database, which is based on physicians’ diagnostic code for Parkinson’s disease and the registration code for Parkinson’s disease^37^. Moreover, the physicians must confirm the patients’ clinical conditions in order to receive copayment reduction for Parkinson’s disease-related medical care and this ensures that this systematic makes the data regarding Parkinson’s disease are reliable^16,17,37^. It should also be emphasized that this study is a large-scale population-based study evaluating the impact of periodontal disease on the development of Parkinson’s disease with a nationwide dataset.

In conclusion, a weak association between periodontitis and Parkinson’s disease was suggested after adjusting for confounding factors from the population-based large-scale cohort of the entire South Korean population.
